# Long non-coding RNA LINC00930 targeting miR-6792-3p/ZBTB16 regulates the proliferation and EMT of pancreatic cancer

**DOI:** 10.1186/s12885-024-12365-9

**Published:** 2024-05-24

**Authors:** Yingqing Mao, Xian Su, Qingsong Guo, Xihao Yao, Qun Zhao, Yibing Guo, Yao Wang, Xiaohong Li, Yuhua Lu

**Affiliations:** 1 Research Center of Clinical Medical, Department of General Surgery, Affiliated Hospital of Nantong University, Medical School of Nantong University, Nantong, 226001 P. R. China; 2 The Sixth People’s Hospital of Nantong, Nantong, 226001 P. R. China; 3 Department of Hepatobiliary Surgery, Shanghai Municipal Hospital of Traditional Chinese Medicine, Shanghai University of Traditional Chinese Medicine, Shanghai, 200000 P. R. China

**Keywords:** LINC00930, miR-6792-3p, ZBTB16, Malignant process, Pancreatic cancer

## Abstract

Emerging evidence suggests the dysregulation of long non-coding RNAs (lncRNAs) involved in pancreatic cancer (PC). However, the function of LINC00930 in PC has not been elaborated. In this study, we found that LINC00930 was significantly down-regulated in PC cell lines and tissues, and associated with tumor size, lymphatic metastasis, TNM stage and poor prognosis. According to the bioinformatics database, the downregulation of LINC00930 was a common event in PC associated with prognosis and EMT. Overexpression of LINC00930 inhibited the aggressive cancer phenotypes including proliferation, metastasis and epithelial-mesenchymal transition (EMT) of PC *in vitro and in vivo.* Bioinformatics and dual-luciferase reporter assay indicated that miR-6792-3p could directly bind to LINC00930. Additionally, the Zinc finger and BTB domain containing 16 (ZBTB16) was significantly declined in PC, which was predicted to be the downstream gene of miR-6792-3p. MiR-6792-3p mimic rescued the decreased proliferation, metastasis and EMT caused by ZBTB16 in PC cells. The LINC00930/miR-6792-3p/ZBTB16 axis was associated with the malignant progression and process of PC. The relative expression of LINC00930 was negatively correlated with the expression of miR-6792-3p and was closely linked with ZBTB16 levels in PC. LINC00930 might serve as a potential prognostic biomarker and therapeutic target for PC.

**Supplementary Information:**

The online version contains supplementary material available at 10.1186/s12885-024-12365-9.

## Introduction

Pancreatic cancer (PC) is a form of exocrine malignancy characterized by high mortality rates and a poor overall prognosis [1]. As of 2021, PC was the seventh most common cause of cancer-associated mortality, with an estimated 466,000 deaths globally [2]. The causes of PC are as yet unknown, but it is related to chronic pancreatitis and diabetes mellitus. Genomic abnormalities include frequent mutations in KRAS, TP53, CDKN2A and SMAD4 [3]. Surgical resection is currently the most effective treatment, especially for patients in the early stages of PC [4]. Unfortunately, most patients are diagnosed at an advanced stage, for the onset of PC is usually hidden and prone to early metastasis [5]. Hence, there is a considerable unmet need to identify novel therapeutic targets, but also to probe the underlying molecular mechanism of PC, enabling more valid treatment regimens to improve the dismal prognosis for patients with this disease [6].

Long non-coding RNAs (lncRNAs), containing over 200 nucleotides, exhibit limited protein-coding activity [7, 8]. Over the past decade, RNAs (LncRNAs) have been found to occupy a significant proportion of the human genome [9]. Accumulating evidence have suggested that lncRNAs constitute a regulatory system, participating in various cellular processes of tumors [10, 11]. LncRNAs can interact with RNAs, proteins and DNA through different molecular mechanisms such as signallings, decoys, guides, scaffolds and miRNA sponges to induce tumor biological behaviors at the transcriptional levels [12, 13]. A wide array of lncRNAs are dysregulated in PC and have been found to regulate tumor cell proliferative, metabolic, and metastatic activity [14]. For instance, LINC00941, which is up-regulated in PC, promotes the progression of pancreatic cancer by competitively binding miR-335-5p to regulate the Rock1-mediated limk1/cofilin-1 signaling pathway [15]. Relatively, LINC01111, as a downregulated lncRNA in PC, up-regulates the level of DUSP1 by inhibiting the expression of miR-3924, leading to the inactivation of SAPK / JNK signal pathway, thus restraining the malignancy of PC [16]. LINC00930, which was first reported in 2022 to be strongly up-regulated in the nasopharyngeal carcinoma(NPC), positively correlated with tumor stage and grade and associated with lymphatic invasion, metastasis, and poor prognosis [17]. Furthermore, targeting LINC00930 during radiotherapy accelerates tumor shrinkage. This suggested that LINC00930 might be a core regulator of cell proliferation and tumorigenesis, and might be a potential therapeutic target of tumors. However, the specific function and detailed mechanism of LINC00930 in PC remains unclear.

Zinc finger and BTB domain containing 16 (ZBTB16), also known as promyelocytic leukemia zinc finger, was first found in patients with acute promyelocytic leukemia and located on chromosome 11q23 in a gene cluster related to the zinc finger family [18]. Recent studies have reported that ZBTB16 is also associated with a variety of digestive system tumors [19]. Emerging literature supports that restoration of ZBTB16 led to induction of G2/M phase arrest and reversal of EMT through antagonizing BCL6/ZBTB27 breast cancer [20].

Herein, we found that the expression of LINC00930 was significantly down-regulated in PC cell lines and tissues, and associated with tumor size, lymphatic metastasis, TNM stage and poor prognosis. The dysregulation of LINC00930 promoted the proliferation, invasion and migration of PC cell in vitro and *in vivo.* Mechanically, LINC00930 modulated the proliferation, metastasis and EMT progression of PC via regulation of miR-26a-5p/ZBTB16 axis. LINC00930 might act as a potential prognostic biomarker and provide a viable new therapeutic target for PC.

## Materials and methods

### Patient samples

Total 36 paired samples of PC and adjacent non-tumorous tissues were collected from patients hospitalized at the First Affiliated Hospital of Nantong University between April 2018 and May 2021. All patients involved in this research had not received any treatment at first diagnosis and informed consents were signed by all of them. Participants who diagnosed with benign tumors after surgery were excluded. Both tumor and non-tumor samples were confirmed by pathological examinations. Tissues that are at least 3 to 5 cm away from the edge of tumor were defined as paracancerous tissues. Each sample was immediately frozen in liquid nitrogen after surgical resection and then stored at -80℃ within 30 min. Besides, this study had obtained the approval from the Ethics Committee of the First Affiliated Hospital of Nantong University.

### The gene expression profiling interactive analysis (GEPIA) dataset

GEPIA (http://gepia.cancer-pku.cn) was used to analyze RNA sequencing materials based on the GTEx and TCGA projects with the normalized processing pipeline. The correlation analysis was completed by TCGA (https://portal.gdc.cancer.gov/), and the drawing was done by Xiantao academic (https://www.xiantaozi.com/). GEPIA is an interactive web server to offer the differential expression analyses on normal and tumor tissues, as well as the access to the profiling of cancer type and pathologic stage, analysis of patient survival, dimensionality reduction and correlation analyses.

### Cell culture

The pancreatic epithelial cell lines HPDE6-C7 and PC (PANC-1, CFPAC‑1, and MIAPaCa-2) were obtained from the Chinese Academy of Sciences Cell Bank (Shanghai, China), and cultured for fewer than 6 months after resuscitation. PANC-1 and HPDE6-C7 cells were maintained in high-glucose DMEM (Gibco, USA) containing 10% FBS (Gibco, USA) and 1% penicillin (Invitrogen, USA). CFPAC1 cells were grown in IMDM (Gibco, USA) containing 10% FBS (Gibco, USA) and 1% penicillin (Invitrogen, USA). MIA-PaCa-2 cells were grown in high -glucose DMEM (Gibco, USA) containing 10% FBS (Gibco, USA), 5% HS (Gibco, USA), and 1% sodium pyruvate and streptomycin (Invitrogen, USA). All cells were cultured in a 5% CO_2_ 37 °C humidified incubator.

Cells were transfected with pcDNA-LINC00930, pcDNA-Vector negative control, miR-6792-3p mimic, or miR-NC negative control (RiboBio, China). Lipofectamine® 3000 (Invitrogen, USA; Thermo Fisher Scientific, USA) was used based on provided directions to transfect cells with appropriate constructs.

### Cell transfection

Transfection procedures were performed according to manufacturer’s protocol. Briefly, PANC-1 and CFPAC-1 cells (10^5^/mL) grown in the 6-well plates for 24 h and then transfected. Hsa-miR-6792-3p mimics and mimics NC were obtained from RiboBio (RiboBio, China) and transfected into PANC-1 and CFPAC-1 cells by riboFECTCP transfection kit (RiboBio, China). Human LINC00930 and ZBTB16 over-expression plasmid and pcDNA3.1-vector were obtained from RiboBio (RiboBio, China).

### CCK-8 assay

Cellular proliferation was assessed in a CCK-8 assay. Cells were transfected with miR-6792-3p mimics, pcDNA-LINC00930, or control constructs prior to plating in 96-well plates at a density of 1000 cells per well. At appropriate time points (24, 48, 72, 96, or 120 h), 10 µl of CCK-8 solution (Dojindo, Japan) was added per well followed by an additional 2 h incubation. Absorbance at 450 nm was then measured via microplate reader (Molecular Devices, USA).

### Wound healing assay

Appropriate CFPAC-1 and PANC-1 cells were cultured in 6-well plates to confluence, at which time a scratch wound was generated using a sterile pipette tip. Non-adherent cells were removed by washing with PBS, and cells were cultured for 48 h in serum-free media. Cells were then imaged via microscopy to quantify wound healing.

### Colony formation assay

Appropriate CFPAC-1 and PANC-1 cells were added to 6-well plates (100/well) and incubated for 14 days, after which colonies were fixed for 30 min with 4% formaldehyde, stained for 20 min with 0.5% crystal violet, and counted. The colonies was determined by measuring the area covered by the colony using Image J analysis software. The average colony size was calculated by summing the areas of all colonies and dividing by the total number of colonies.

### Transwell assay

At 48 h post-transfection, cells were harvested and resuspended at 1.0 × 10^5^ cells/mL in serum-free media. These cells were then added to the upper chamber of a Transwell insert, with culture media being added to the lower chamber. Cells were then incubated for 36 h, after which the insert was rinsed thrice with PBS, fixed for 15 min with methanol, and cells in five random fields of view per insert were counted to analyze cellular migration. The photographs of cell migration were collected under a 200-fold microscope, and cell number was calculated in 3 visual fields that were randomly selected, with the average value considered as the numbers of cells penetrating the membrane. The experiment was repeatedly conducted for 3 times. Image J software was used to assess the cell migration capacity.

### qRT-PCR

Trizol (Invitrogen, USA) was used to extract RNA from cells based on provided directions. A High Capacity cDNA Synthesis kit (Applied Biosystems, USA ) was used to synthesize DNA. All qPCR reactions were performed with a SYBR Premix Ex Taq kit (Takara Biotechnology, China) and an Applied Biosystems 7500 Real-Time PCR instrument (Thermo Fisher Scientific, UK). MiRNA quantification: Bulge-loop™ miRNA qPT-PCR Primer Sets were designed by RiboBio (Guangzhou, China). U6 and 18 S rRNA served as controls for miRNA and lncRNA expression, respectively. QRT -PCR analysis was performed using the Power SYBR Green PCR Master Mix (RiboBio, China) with primer sequences in Table 1.

**Table 1:** qRT-PCR primers for the detected genes

Genes	Forward primer	Reverse primer
U6	5’-CTCGCTTCGGCAGCACA-3’	5’-AACGCTTCACGAATTTGCGT-3’
LINC00930	5’-CCTTACATCTGGGCTCCAT-3’	5’-CGGCTGGTTTACAACTGAC-3’
ZBTB16	5’-GGACAGTTTGATGACCATAGGA-3’	5’-CTTTGCCTCTTTCCTCAACCTT-3’
miR-6792-3p	5’-CGCTCCTCCACAGCCCCT-3’	5’-AGTGCAGGGTCCGAGGTATT-3’
GAPDH	5’-CTCGCTTCGGCAGCACA-3’	5’-AACGCTTCACGAATTTGCGT-3’

### Dual-luciferase reporter assays

The miRWalk database was used to identify miR-6792-3p as a putative LINC00930 target. Wild-type (WT) and mutant (MUT) versions of the predicted miR-6792-3p binding site from LINC00930 were amplified and cloned into the pmir-GLO vector (RiboBio, China). Recombinant LINC00930-WT and LINC00930-MUT primers were co-transfected into PC cells along with miR-6792-3p mimic or NC mimic control. At 48 h post-transfection, a Dual-Luciferase Reporter Assay System (Promega, USA) was used based on provided directions.

### Xenograft model assays

Female BALB/c nude mice (6-weeks-old) from the Laboratory Animal Center of Nantong University were housed under specific pathogen-free conditions at the Nantong University animal care facility. A total of 16 BALB/c nude mice were used, eight in each group. Mice were subcutaneously implanted in the left axilla with 100 µl of PANC-1 or CFPAC-1 cells (1 × 10^6^ cells) following pcDNA-Vector or pcDNA-LINC00930 transfection. Every three days, vernier calipers were used to measure the size of the xenograft tumor, and the volume of the tumor was determined by applying the following formula: (length×width^2)/2. After 24 days, mice were intraperitoneally injected with 3% sodium pentobarbital (30 mg/kg) and euthanized via cervical dislocation. The Animal Center of the Medical College of Nantong University guidelines for the care and use of experimental animals were used to conduct the present studies, which received approval from the Animal Center of the Medical College of Nantong University.

### Immunohistochemical staining

Ki67 protein levels were measured via immunohistochemical (IHC) staining. Briefly, paraffin-embedded tumor tissue Sect. (4 μm) were stained overnight with appropriate primary antibodies (1:1000; Cat No. 15,580, Abcam, UK) at 4 °C. Sections were then stained with HRP-conjugated secondary antibodies(1:200; Cat No. 6721, Abcam, UK) for 30 min at room temperature, followed by 3, 3′-diaminobenzidine (ABclonal, China) staining and imaging using a Leica DMi8 microscope.

### Western blot analysis

Cells were lysed in RIPA reagent (Beyotime Biotechnology, China) and resolved by SDS-PAGE electrophoresis. Proteins were then transferred to PVDF membranes (Millipore, USA), blocked with a quick sealant, and incubated with the following rabbit primary antibodies for overnight at 4 °C (all from Abcam, UK): N-cadherin (1:5000; Cat No. ab76011), E-cadherin (1:5000; Cat No. ab213103), Vimentin (1:20000; Cat No. ab20346), β-actin (1:2000; Cat No. ab8227), Bcl-2 (1:1000; Cat No. ab32124), and ZBTB16 (1:5000; Cat No. DF2611, Affinity BioReagents, USA). After washing with TBST three times, the membranes were incubated with horseradish peroxidase (HRP)-conjugated secondary antibodies (1:50000; Cat No. ab205718) for 1 h at room temperature. Bands were visualized with a ChemiDoc XRS imaging system (Bio-Rad, USA).

### Statistical analysis

SPSS 19.0 (IBM Corp, USA) was utilized for all statistical testing. Data were compared using two-tailed Student’s t-tests or one-way ANOVAs, and experiments were repeated three or more times. The difference in LINC00930/miR-6792-3p expression between PC and matched normal tissues were examined using the paired t-test. Relationships between LINC00930/miR-6792-3p expression and clinicopathological characteristics were assessed via Pearson χ2 tests. A value of *p* < 0.05 was considered statistically significant.

## Results

### LINC00930 was downregulated in PC and associated with poor survival

**Fig. 1:**
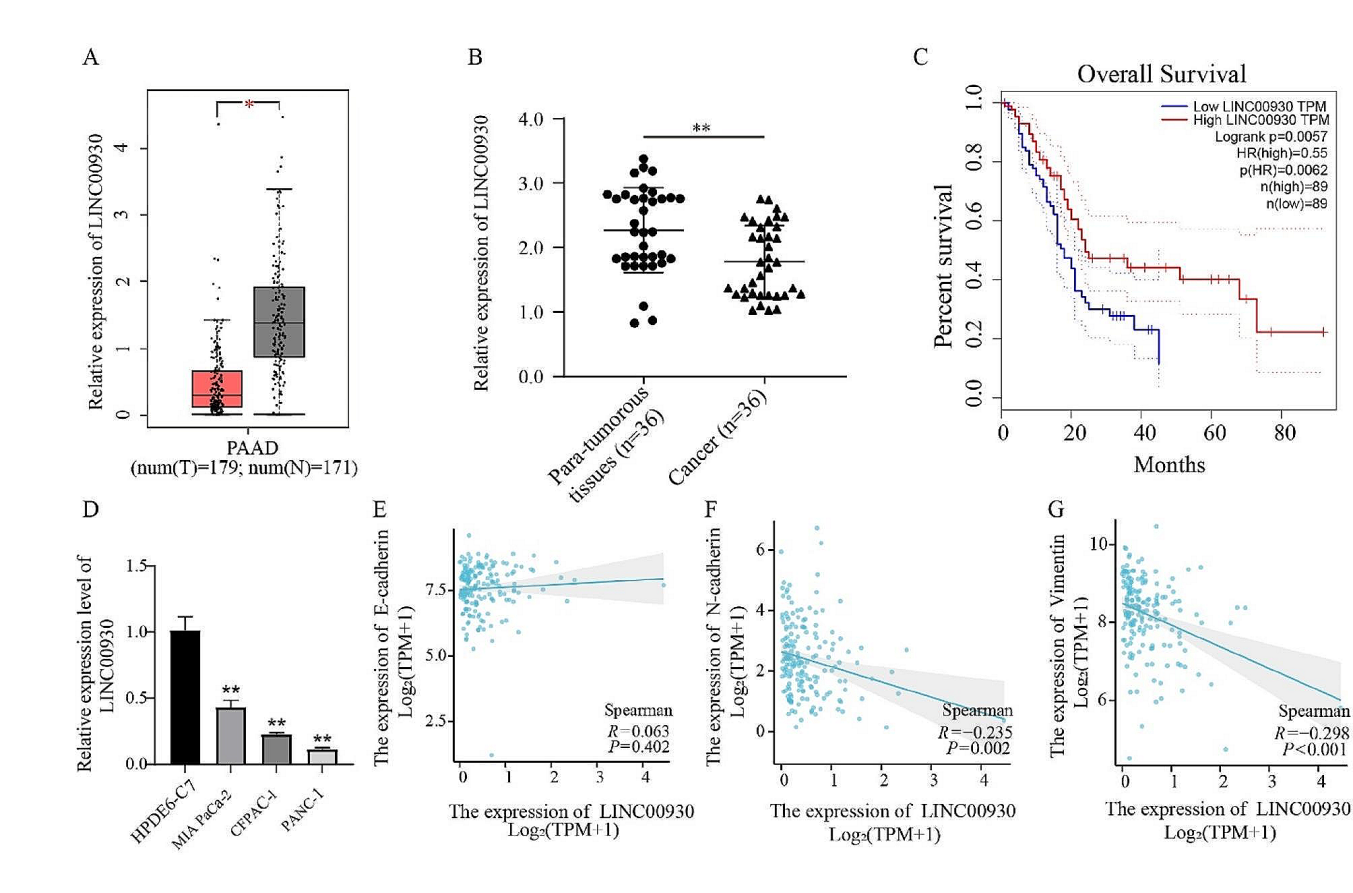
LINC00930 was down-regulated in PC and associated with poor survival. (**A**) LINC00930 expression in PC tissues from the GEPIA database. (**B**) QRT-PCR analysis of LINC00930 expression in 36 pairs of PC tissues and paracancerous tissues. (**C**) Association of LINC00930 expression with overall survival of PC patients. (**D**) LINC00930 expression levels in HPDE6-C7 cells and the PC cell lines. (**E**-**G**) Correlation analysis between LINC00930 and EMT related molecules. E-cadherin, N-cadherin, and Vimentin. Data are means ± SD, *p < 0.05, **p < 0.01, ***p < 0.001

The expression of LINC00930 was identified by the GEPIA database, revealing that LINC00930 levels were lower in PC tissues compared with para-tumorous tissues (Fig. 1A). For the purpose of investigating the role of LINC00930 in PC, qRT-PCR analysis was performed to detect the expression of LINC00930 in PC tumor tissues and adjacent normal ones from 36 patients. As evident from Fig. 1B, a significantly increased expression level of LINC00930 was observed in para-tumorous tissues, compared with matched malignant ones. To determine the clinical role of LINC00930, we analyzed the correlation between LINC00930 levels and the clinicopathological features of PC patients. High LINC00930 expression levels were associated with tumor diameter in PC tissues, lymphatic metastasis and TNM stage. However, there was no significant correlation between LINC00930 levels and gender, age or differentiation degree (Table 2). Kaplan-Meier analysis indicated that lower LINC00930 expression levels were associated with poorer survival outcomes (Fig. 1C). In addition, LINC00930 expression was dramatically downregulated in PC cell lines, particularly the CFPAC-1 and PANC-1, compared with pancreatic epithelial cell line HPDE6-C7 (Fig. 1D). EMT has been reported to contribute to the malignant processes as well as relate strongly to poor prognosis of PC [21]. Hence, we investigated the relationship between LINC00930 and EMT, and found that LINC00930 was negatively correlated with EMT-related genes N-cadherin and Vimentin but not E-cadherin by TCGA (Fig. 1E-G)Together, these results suggested LINC00930 may as a regulator of development and progression in PC.

**Table 2:** Relationships between LINC00930 expression and clinicopathological characteristics

Clinicopathological characteristics		*N*	Low expression	High expression	*P* value	χ^2^
**Gender**					0.738	0.111
Male		19	10	9		
Female		17	8	9		
**Age (years)**					0.735	0.114
≤ 60		15	7	8		
>60		21	11	10		
**Tumor diameter (cm)**					0.002*	9.257
≤ 4		21	6	15		
>4		15	12	3		
**Lymphatic metastasis**					0.018*	5.6
Absent		21	7	14		
Present		15	11	4		
**TNM stage**					0.019*	4.461
I-II		19	6	13		
III-IV		17	12	5		
**Grade of differentiation**					0.782	0.493
High		5	3	2		
Middle		22	10	12		
Low		9	5	4		

*p < 0.05 was considered statistically significant

### LINC00930 inhibited proliferation, migration and invasion of PC cells

**Fig. 2:**
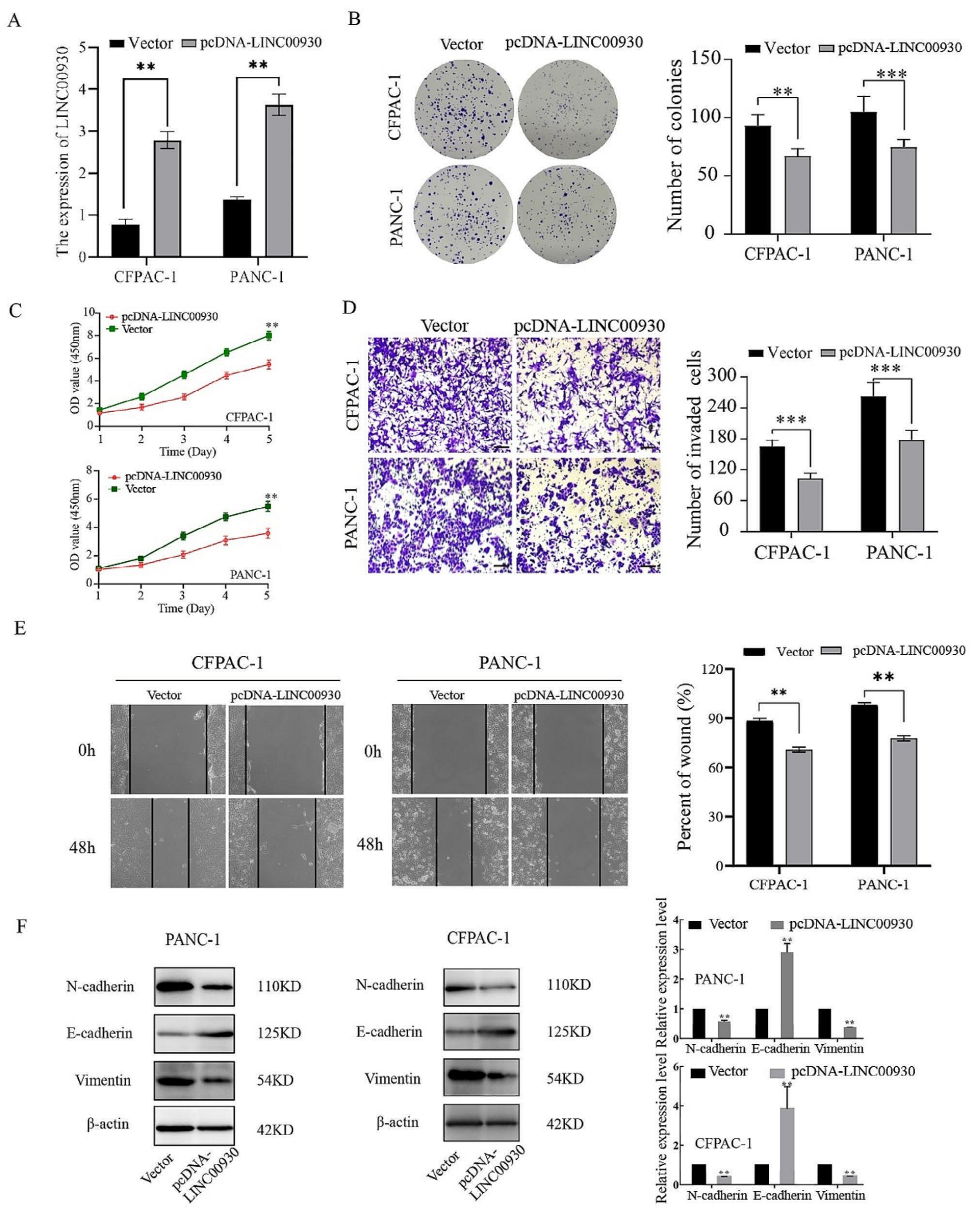
LINC00930 inhibited proliferation, migration and EMT of PC cells in vitro. (**A**) Validation of overexpression efficiency in PANC-1 and CFPAC‑1 cells by qRT-PCR. (**B**-**E**) The proliferation, migration, invasion, and colony formation abilities of PANC-1 and CFPAC‑1 cells transfected with pcDNA-LINC00930 or pcDNA-Vector were correspondingly estimated by colony formation, CCK-8, transwell assays and wound healing (Scale bar = 100 μm). (**F**) Western blot were performed to analyse the expressions of EMT biomarkers with or without LINC00930 overexpressing. The gels are cropped due to the difference in molecular weights. The gels are cropped due to the difference in molecular weights, and the samples derive from the same experiment and that gels/blots were processed in parallel. Data are means ± SD, *p < 0.05, **p < 0.01, ***p < 0.001

Next, LINC00930 was overexpressed in PANC-1 and CFPAC-1 via pcDNA transfection (Fig. 2A). Subsequent colony formation and CCK-8 assays revealed that such overexpression impaired the proliferative activity of both tested cell lines relative to vector control cells (Fig. 2B, C). PC cell invasion and migration were also markedly impaired upon LINC00930 overexpression, as observed in transwell invasion and wound healing assays (Fig. 2D, E). Increasing evidence shows that the EMT plays a pivotal role in metastasis processes of PC, and a correlation between LINC00930 and EMT was found in the TCGA database, meanwhile, western blot analysis showed that the overexpression of LINC00930 led to an increase in the epithelial marker E-cadherin, and suppressed expression of the mesenchymal markers, N-cadherin and Vimentin, at the protein level (Fig. 2F). The overexpression of LINC00930 could thus markedly ablate the malignancy of PC cells in vitro.

### LINC00930 suppressed the growth of PC xenograft tumor in vivo

**Fig. 3:**
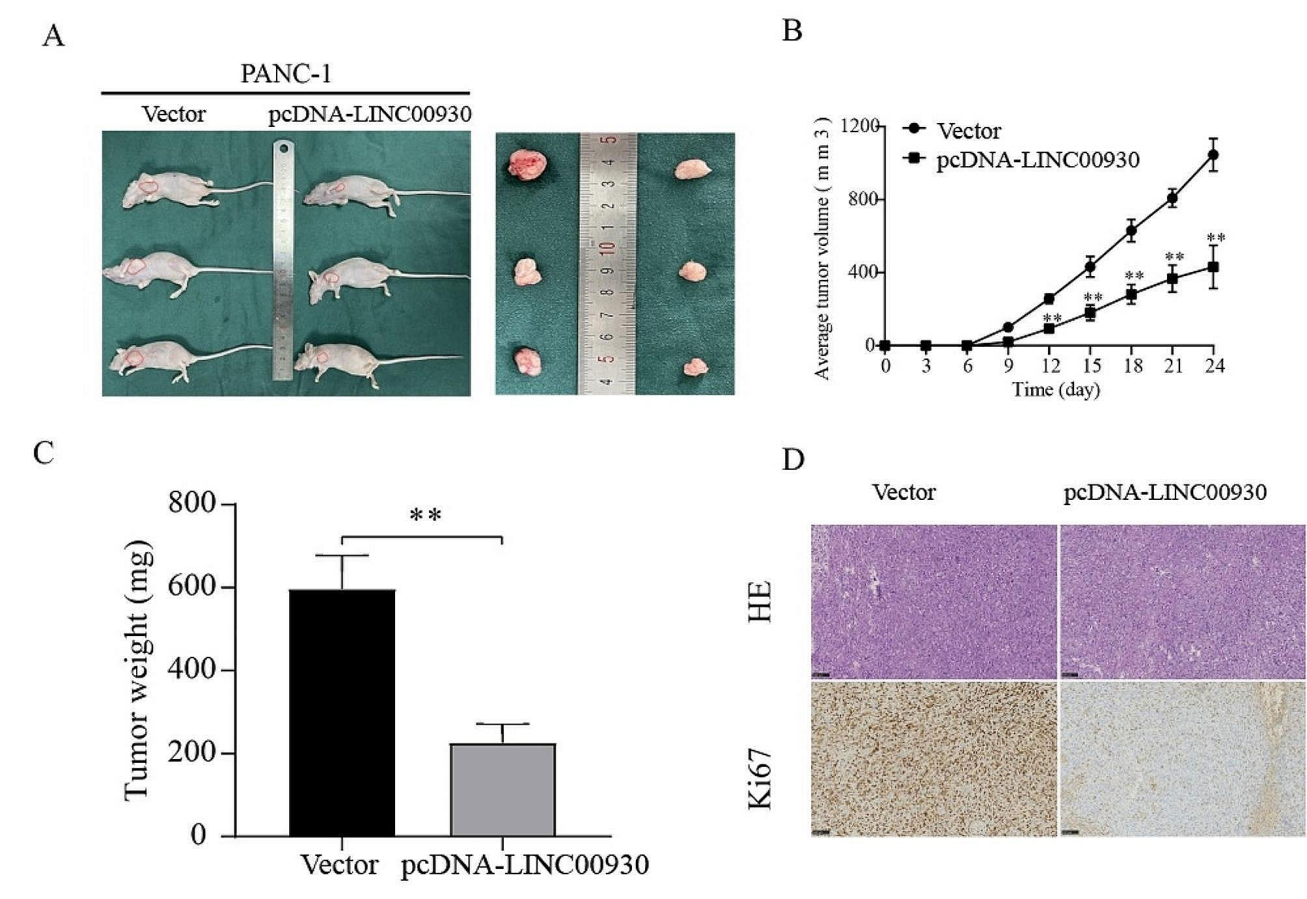
LINC00930 suppressed the growth of PC xenograft tumor in vivo. (**A**) Representative images of subcutaneous xenograft tumors. (**B**-**C**) Tumor volumes and weights were measured at the indicated times. (**D**) H&E and Ki67 staining of tumor tissues of the two xenograft groups. Data are means ± SD, *p < 0.05, **p < 0.01, ***p < 0.001

To explore the effect of LINC00930 on the tumorigenesis of PC in vivo, PC cells were inoculated into nude mice in the left axilla after stably transfected with pcDNA-LINC00930 or vector, and we observed the growth rate of tumors in the pcDNA‐LINC00930 group was substantially suppressed compared with the control group (Fig. 3A, B). Consistent changes in tumor weight were also observed between these two groups (Fig. 3C). Lower levels of Ki-67 expression were also observed in the pcDNA‐LINC00930 transfection group via IHC staining (Fig. 3D). Overall, these results confirmed that overexpression of LINC00930 suppressed tumor growth of PC.

### LINC00930 directly interacted with mir-6792-3p in PC cells

**Fig. 4:**
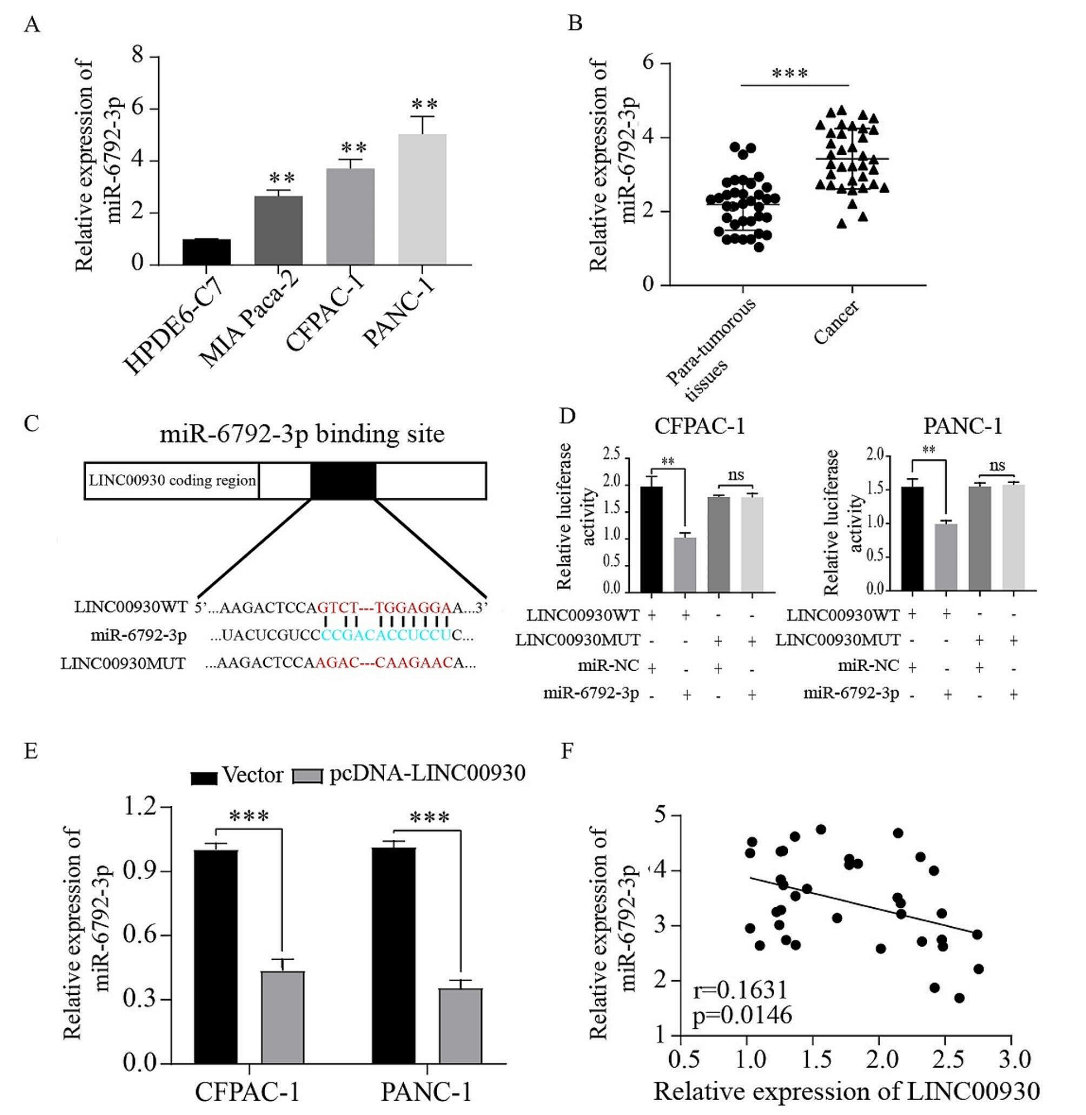
LINC00930 directly interacted with miR-6792-3p in PC cells. (**A**, **B**) MiR-6792-3p expressions in PC cell lines and PC tissues. (**C**) The predicted binding sites of miR-6792-3p to the LINC00930 sequence. (**D**) Luciferase reporter assay for determining whether miR-6792-3p could bind to LINC00930. (**E**-**F**) Correlations between LINC00930 and miR-6792-3p expression in PC cell lines and PC tissues. Data are means ± SD, *p < 0.05, **p < 0.01, ***p < 0.001

MiR-6792-3p was predicted to be a potential target of LINC00930 by online database LNCipedia (Fig. 4C). MiR-6792-3p is highly expressed in gastric cancer tissues and correlated with the progression and poor prognosis [22]. QRT-PCR analysis revealed that the relative expression level of miR-6792-3p was significantly up-regulated in PC cells and tissues compared with pancreatic cells and para-tumorous tissues (Fig. 4A, B). Dual-luciferase reporter assays confirmed that miR-6792-3p could bind directly to the LINC00930 (Fig. 4C, D). Additionally, the expression levels of miR-6792-3p were decreased in PC cells transfected with pcDNA-LINC00930 (Fig. 4E). Similarly, the expression of miR-6792-3p was negatively correlated with the expression of LINC00930 in PC tissues (Fig. 4F). Therefore, we confirmed that LINC00930 was directly interacts with miR-6792-3p in PC cells.

### LINC00930 regulated malignant phenotype of PC cell in a mir-6792-3p-dependent manner

**Fig. 5:**
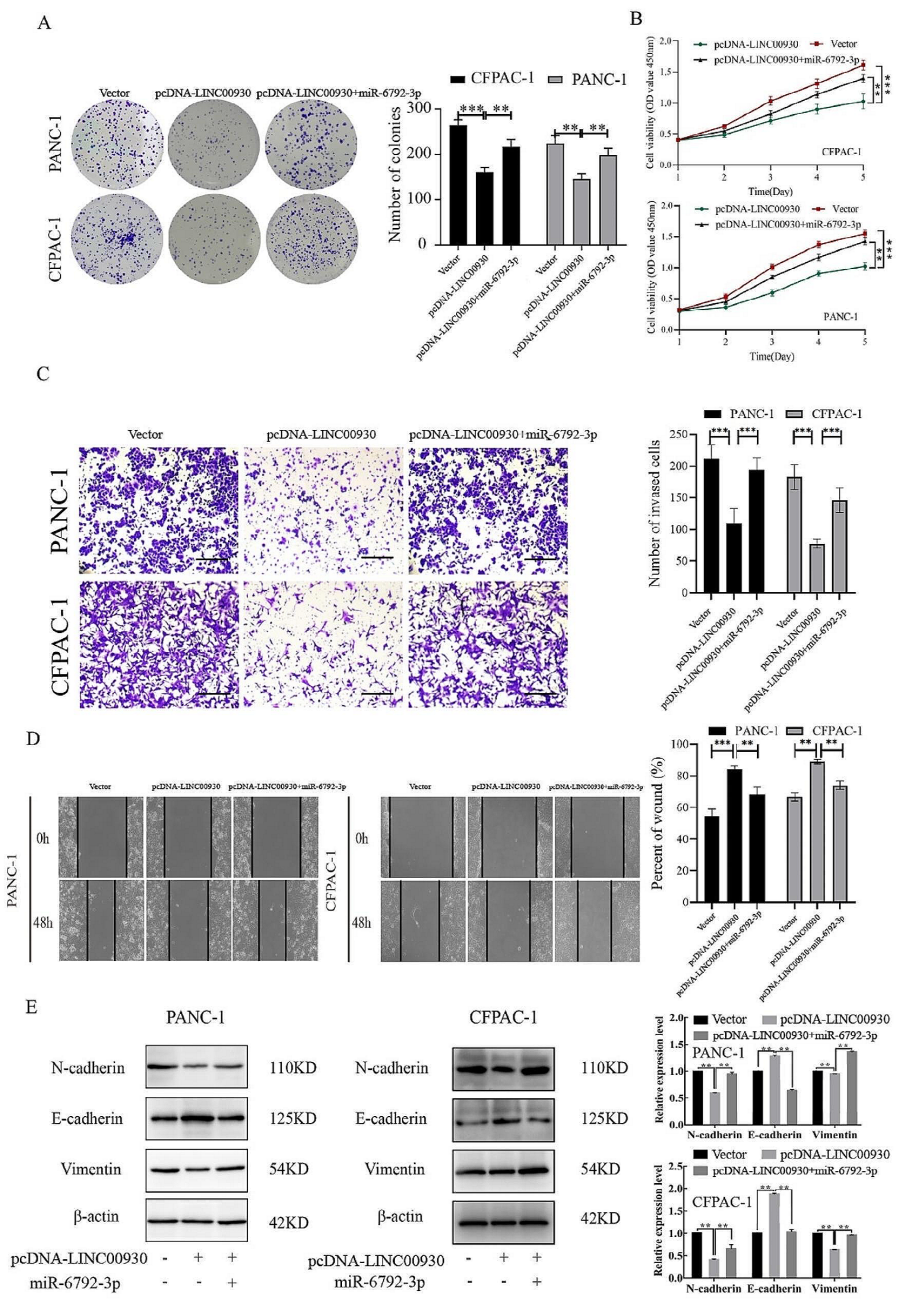
LINC00930 regulated malignant phenotype of PC cell in a miR-6792-3p-dependent manner. (**A**-**B**) Proliferation of PANC-1 and CFPAC-1 cells overexpressing both LINC00930 and miR-6792-3p was determined by CCK8 assays and colony formation assays. (**C**-**D**) The migration and invasion abilities of PANC-1 and CFPAC-1 cells co-transfection LINC00930 and miR-6792-3p mimics were evaluated by transwell and wounding healing assays (Scale bar = 100 μm). (**E**) Western blot analysis of the expression of EMT markers N-cadherin, E-cadherin and Vimentin after co-transfection of LINC00930 and ZBTB16 in PANC-1 and CFPAC-1 cells. The gels are cropped due to the difference in molecular weights, and the samples derive from the same experiment and that gels/blots were processed in parallel. Data are means ± SD, *p < 0.05, **p < 0.01, ***p < 0.001

To investigate the functional relevance of LINC00930 targeting by miR-6792-3p, we assessed whether miR-6792-3p overexpression could rescue the inhibitory effects of LINC00930 on proliferation, migration, invasion and EMT activity. PANC-1 and CFPAC-1 cells were co-transfected with both pcDNA-LINC00930 and miR-6792-3p mimic. PC cells co-transfection with pcDNA-LINC00930 and miR-6792-3p were found to reverse the malignant phenotype induced by pcDNA-LINC00930 in subsequent colony formation, CCK-8, migration, and transwell invasion assay (Fig. 5A-D). Since our previous study showed that LINC00930 could regulate the EMT-related protein, we investigated whether miR-6792-3p was required for LINC00930 to regulate EMT activity in PC cells. Western blot analysis showed that miR-6792-3p rescued the inhibitory effect of LINC00930 on EMT-related protein (Fig. 5E). Together, these data suggested that LINC00930 could regulate the malignant phenotype via miR-6792-3p.

### Mir-6792-3p promoted proliferation, migration and EMT via ZBTB16 in PC

**Fig. 6:**
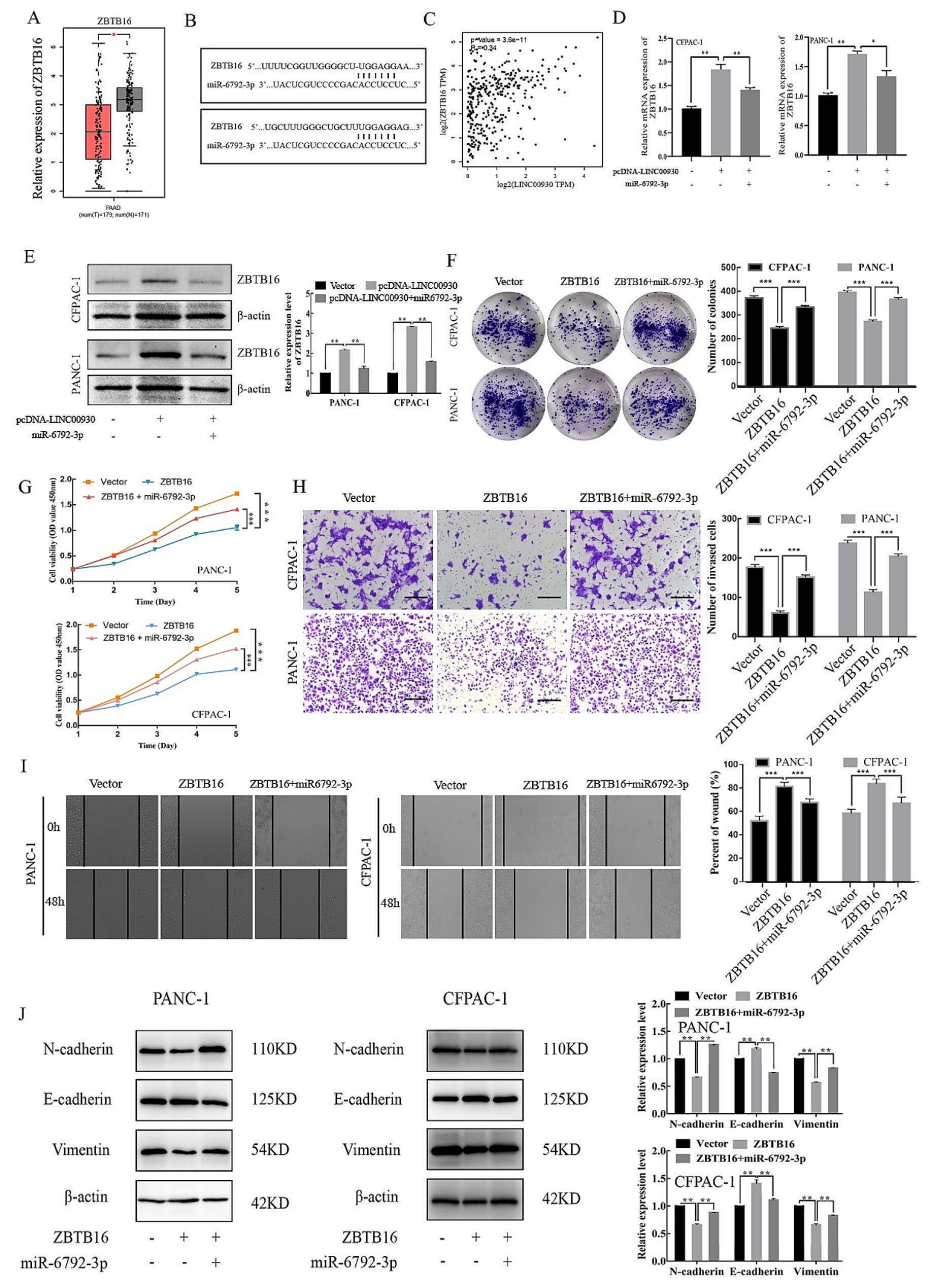
miR-6792-3p promoted proliferation, migration and EMT via ZBTB16 in PC. (**A**) The expression of ZBTB16 in PC tissues and paratumor tissues in GEPIA. (**B**) The miR-6792-3p binding site predicted in the sequence of ZBTB16. (**C**) The relationship between LINC00930 and ZBTB16 in GEPIA. (**D**-**E**) qRT‐PCR and western blot were used to detect the expression of ZBTB16 after co-transfected of LINC00930 and miR‐6792‐3p. (**F**-**I**) The proliferation, invasion and migration of PC cells co-transfected of ZBTB16 and miR-6792-3p were detected by colony formation, CCK8, transwell and wound healing assay (Scale bar = 100 μm). (**J**) Western blot was performed to detect the expression of EMT markers N-cadherin, E-cadherin, and Vimentin after the co-transfection of ZBTB16 and miR-6792-3p in PANC-1 and CFPAC-1 cells. The gels are cropped due to the difference in molecular weights, and the samples derive from the same experiment and that gels/blots were processed in parallel. Data are means ± SD, *p < 0.05, **p < 0.01, ***p < 0.001

It is reported that Zinc finger and BTB domain containing 16 (ZBTB16) inhibited breast cancer proliferation and metastasis through upregulating ZBTB28 and antagonizing BCL6/ZBTB27 [20]. According to GEPIA database, ZBTB16 exhibited low-expression in tumor tissue and was positively correlated with LINC00930. In addition, ZBTB16 was predicted to be the downstream of miR-6792-3p in two potential binding sites by TargetScan database (Fig. 6A-C). Moreover, the expression of ZBTB16 was reversed after co-transfection of LINC00930 and miR-6792-3p mimic (Fig. 6D-E). In addition, we also found that exogenous expression of miR-6792-3p abolished the inhibition of proliferation, invasion and migration induced by ZBTB16 in PC cells (Fig. 6F-I). Next, we measured the levels of EMT-related proteins by western blotting. Results showed that ZBTB16 overexpression led to increased expression of E-cadherin but decreased expression of N-cadherin and vimentin in PC cells, indicating that the epithelial cells acquired mesenchymal properties. Nevertheless, these mesenchymal properties were reversed by co-overexpression of miR-6792-3p (Fig. 6J). Our data suggested that miR-6792-3p could promote proliferation, migration and EMT by counteracting the effects of ZBTB16 in PC.

**Fig. 7:**
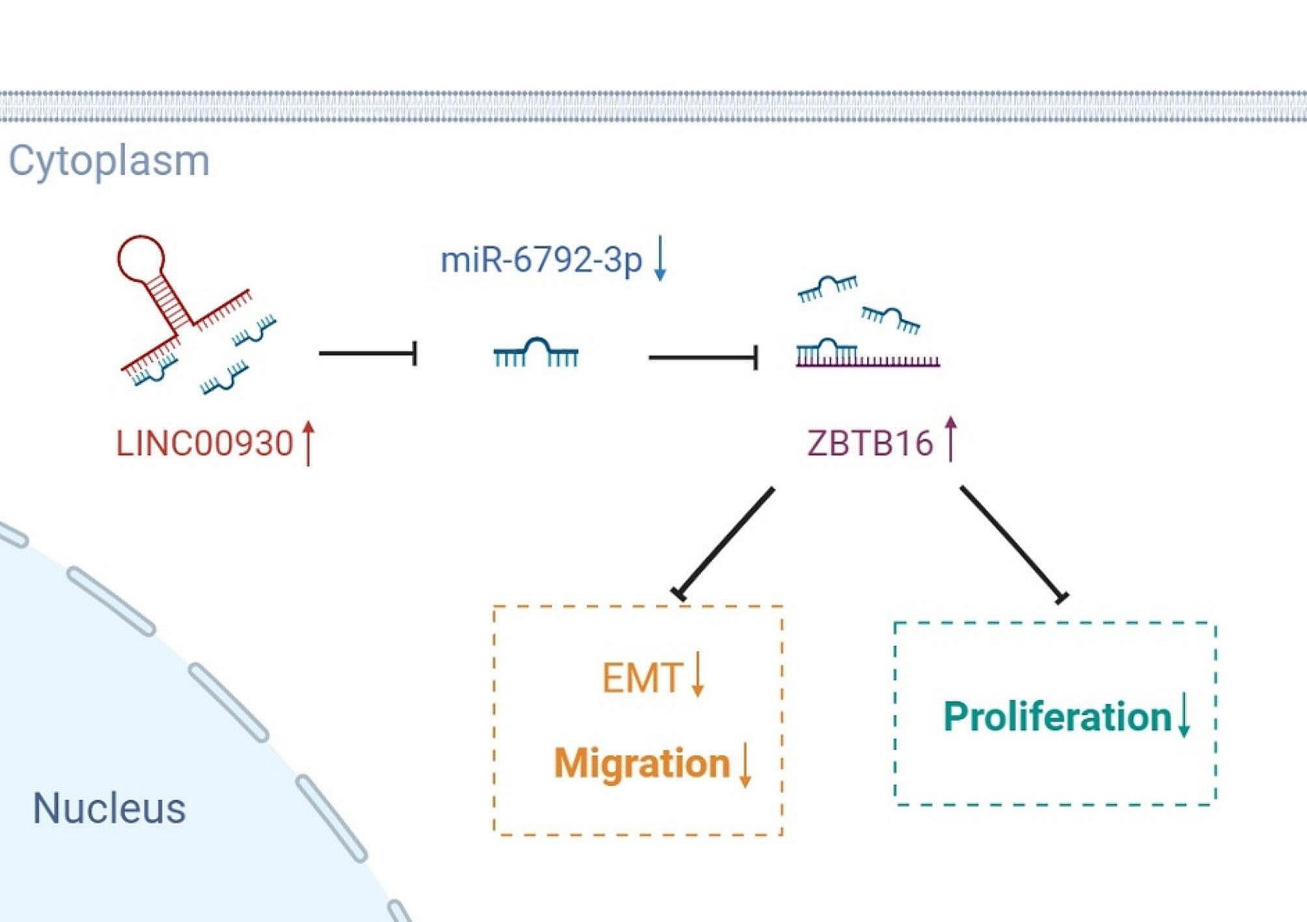
Schematic diagram revealed the regulatory mechanisms of the LINC00930/miR-6792-3p/ZBTB16 axis on migration, proliferation and EMT activity in PC cells

## Discussion

PC is the most aggressive and deadliest form of cancer among humans [23]. The effect of surgical treatment of pancreatic cancer is limited by the clinical stage of PC [24, 25]. Given the current shortage of specificity in diagnosis and targeted therapy, the discovery of prognostic biomarkers and therapeutic targets for PC is crucial [26–28]. In our study, we found that LINC00930 was significantly down-regulated in PC cell lines and tissues, and inhibited the proliferation, metastasis and EMT of PC in vitro and in vivo. We further revealed that miR-6792-3p could directly bind to LINC00930, meanwhile, overexpression of miR-6792-3p could reverse the inhibition of proliferation, migration and invasion induced by LINC00930. Our study demonstrated that LINC00930 could regulate malignant phenotype via miR-6792-3p/ZBTB16 axis, and might serve as a therapeutic target for PC.

A broad spectrum of evidence indicates that lncRNAs play integral roles in regulating oncogenesis and malignant progression of PC [29, 30]. For instance, the lncRNA RUNX1-IT1, as a trans-acting factor, enhances the malignant process of PC by recruiting RUNX1 to the C-FOS gene promoter [31]. LINC00976 up-regulates the expression of OTUD7B by targeting downstream miR-137, which mediates EGFR and MAPK signaling pathways, thus increases the proliferation and invasion of PC cells [32]. LINC00930 was first reported in nasopharyngeal carcinoma [17], where it binds the RBBP5 and GCN5 complexes to the PFKFB3 promoter, elevates H3K4 trimethylation and H3K9 acetylation levels, transactivating PFKFB3, thereby promoting glycolytic flux and cell cycle progression. Furthermore, targeting LINC00930 and PFKFB3 during radiotherapy accelerates tumor shrinkage. Here, we found that the expression of LINC00930 was down-regulated in PC tissues, which was closely related to clinicopathological features, including tumor size, lymphatic metastasis, TNM stage and poor prognosis (Fig. 1). And LINC00930 decelerated the malignant phenotype of PC by loss- and gain-function assays both in vitro and in vivo (Fig. 2 and Fig. 3). Our findings indicated that LINC00930 was a mediator of tumor malignancy, highlighting the potential of LINC00930 as a therapeutic approach in PC. The increasing prevalence of lncRNAs with critical functions in PC has been ascertained in recent years, and most of them majorly dominate a multi-step process of cancer development including proliferation, migration, invasion and EMT [33–35].

Functions of long non-coding RNA indirectly affect gene expression by binding and isolating specific miRNA [36–38]. This competing endogenous RNA mechanism is directly related in many carcinogenic environments [39, 40]. Bioinformatics predicted a binding site between miR-6792-3p and LINC00930, and the interaction of LINC00930 with miR-6792-3p was confirmed by dual luciferase assay (Fig. 4). Yu et al. found aberrant expression of miR-6792-3p in circulating and tissue samples, which was closely associated with clinicopathology, survival time and poor prognosis in gastric cancer [41]. Luo et al. further found that circCCDC9, as the “ceRNA” of miR-6792-3p, alleviated the inhibitory effect of miR-6792-3p on its target CAV1, thereby inhibiting the tumorigenesis of GC [42]. Overexpression LINC00930 inhibited proliferation, migration, invasion and EMT activity of PC cells, which could be reversed by co-transfection with miR-6792-3p mimic (Fig. 5). This suggested that miR-6792-3p might be the downstream of LINC00930, which regulated the progression and EMT of PC. Mechanistically, lncRNAs can act as ceRNAs that ‘sponge’ microRNAs, bind with some EMT-related proteins, or directly regulate genes located physically nearby [43, 44].

Zinc finger and BTB domain containing 16 (ZBTB16), also known as promyelocytic leukemia zinc finger, was first found in patients with acute promyelocytic leukemia and is located on chromosome 11q23 in a gene cluster related to the zinc finger family [18, 45, 46]. Recent studies have revealed that ZBTB16 is also associated with various digestive system tumors [47]. We found ZBTB16 bound to miR-6792-3p in two potential sites by TargetScan database (Fig. 6). The malignant progression and EMT activity were significantly reduced in PC cells by overexpression of ZBTB16, which could be abolished by exogenous expression of miR-6792-3p. Moreover, overexpression LINC00930 could reverse the inhibition of miR-6792-3p on ZBTB16 at transcriptional and posttranslational levels. These results indicated that LINC00930 might regulate the malignant process and EMT activity cells through the miR-6792-3p/ZBTB16 axis in PC. ZBTB16 functions as a tumor suppressor through upregulating ZBTB28 and antagonizing BCL6, leading to inhibition of migration and invasion, reversal of EMT, and suppression of cell proliferation in breast cancer [20]. In pancreatic cancer, EMT status of patient-derived tumor specimens determined by the expression of epithelial and mesenchymal markers and EMT-associated transcription factors was a strong predictor of advanced tumor stage, lymphatic invasion, mortality and recurrence [48–50]. Taken together, LINC00930 modulated the malignant progression by regulating ZBTB16 in PC.

## Conclusions

We identified LINC00930 as a crucial lncRNA downregulated in PC, which is inversely correlated with tumor size, lymphatic metastasis, TNM stage and poor prognosis. Moreover, LINC00930 inhibited the proliferation, migration, invasion and EMT process of PC through miR-6792-3p/ZBTB16. Collectively, our study highlighted that LINC00930/miR-6792-3p/ZBTB16 established the network modulating the malignant progression of PC cells, and might be an attractive prognostic predictor for PC and worthy to be deeply explored (Fig. 7).

### Electronic supplementary material

Below is the link to the electronic supplementary material.

Supplementary Material 1

## Data Availability

All data included in this study are available upon request by contact with the corresponding author.
