# Supplementary material for: Long non-coding RNA LINC00930 targeting miR-6792-3p/ZBTB16 regulates the proliferation and EMT of pancreatic cancer
Source: BMC Cancer. 2024 May 24;24:638. doi: 10.1186/s12885-024-12365-9 (PMC11127394; doi:10.1186/s12885-024-12365-9)
Supplement: Supplementary file 1 — Supplementary Material 1 [file 12885_2024_12365_MOESM1_ESM.pdf]

Cropped blots in main paper Original blots in supplementary information

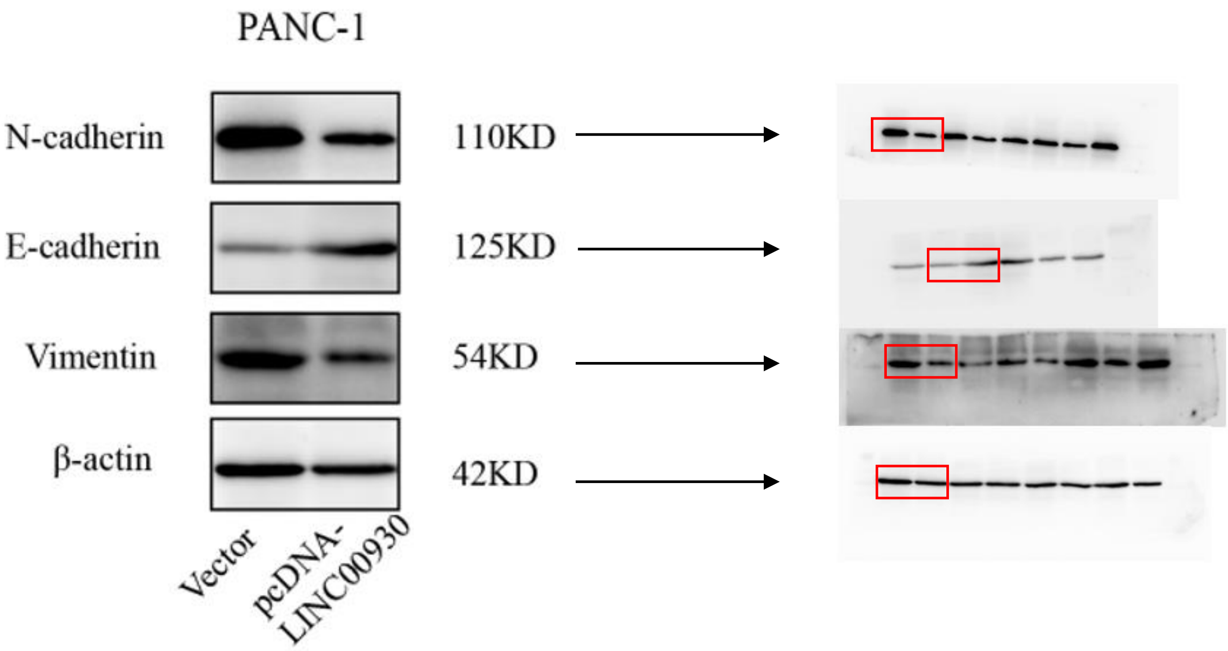

Cropped blots in main paper

Original blots in supplementary information

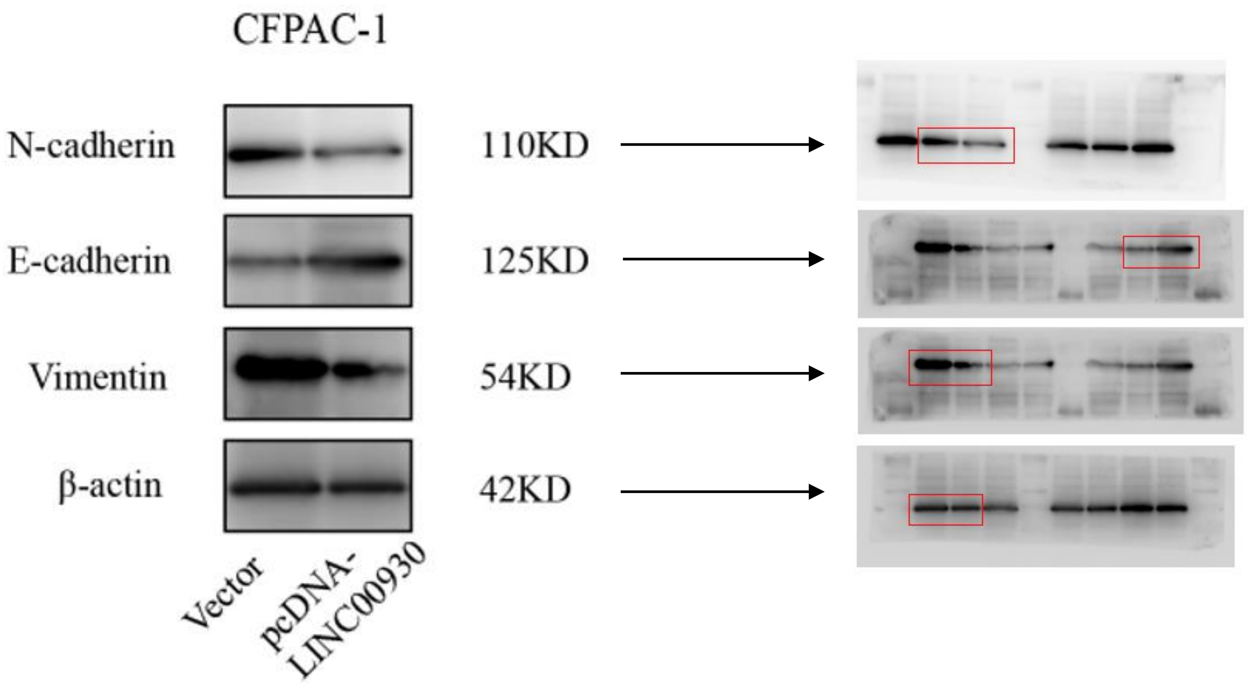

Cropped blots in main paper

Original blots in supplementary information

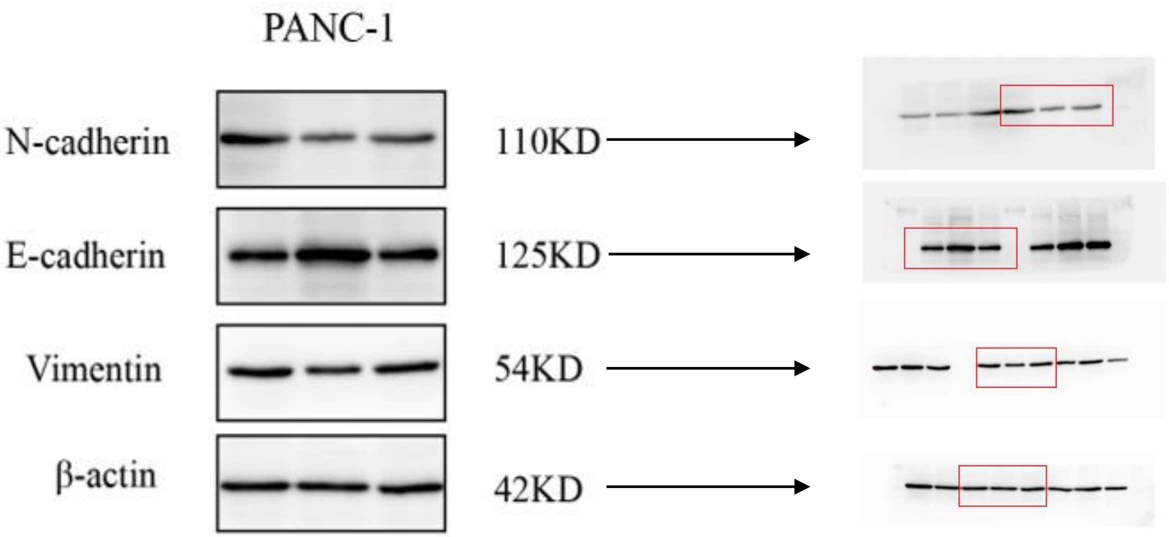

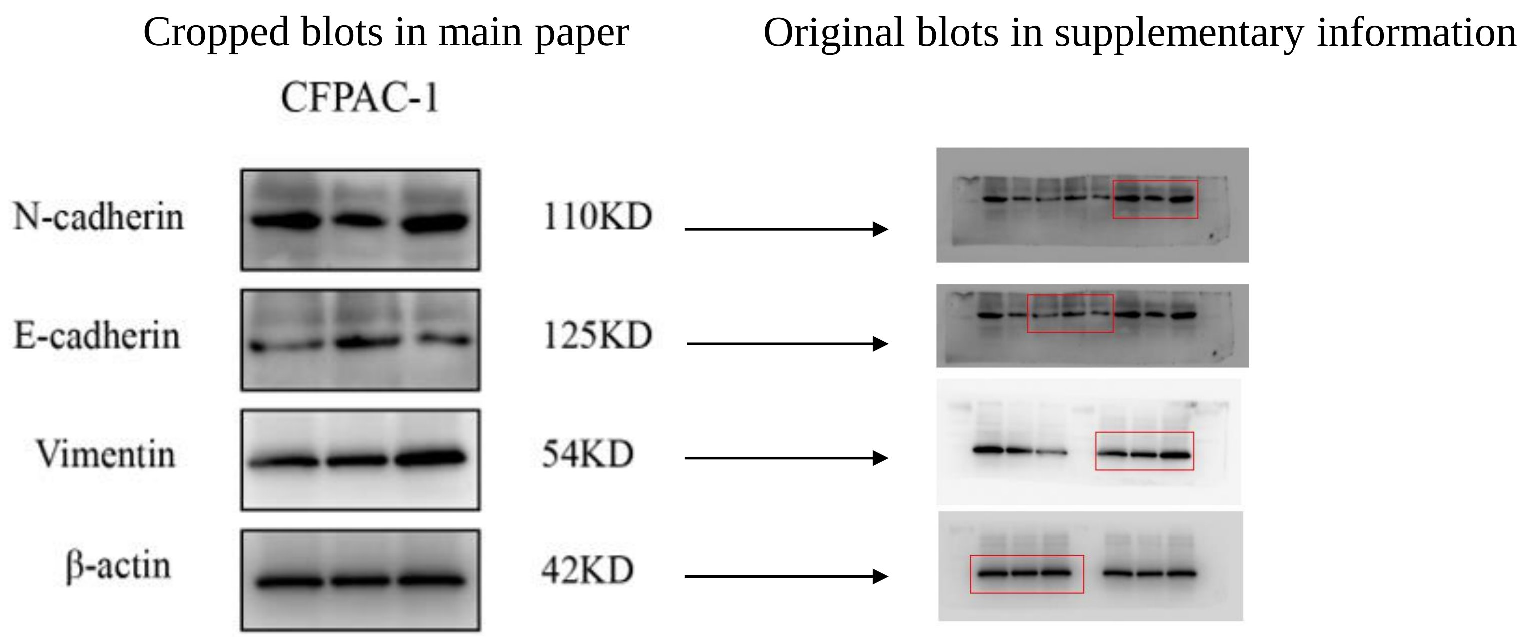
Cropped blots in main paper
Original blots in supplementary information
CFPAC-1
N-cadherin
E-cadherin
Vimentin
β-actin
110KD
125KD
54KD
42KD

Cropped blots in main paper | Original blots in supplementary information

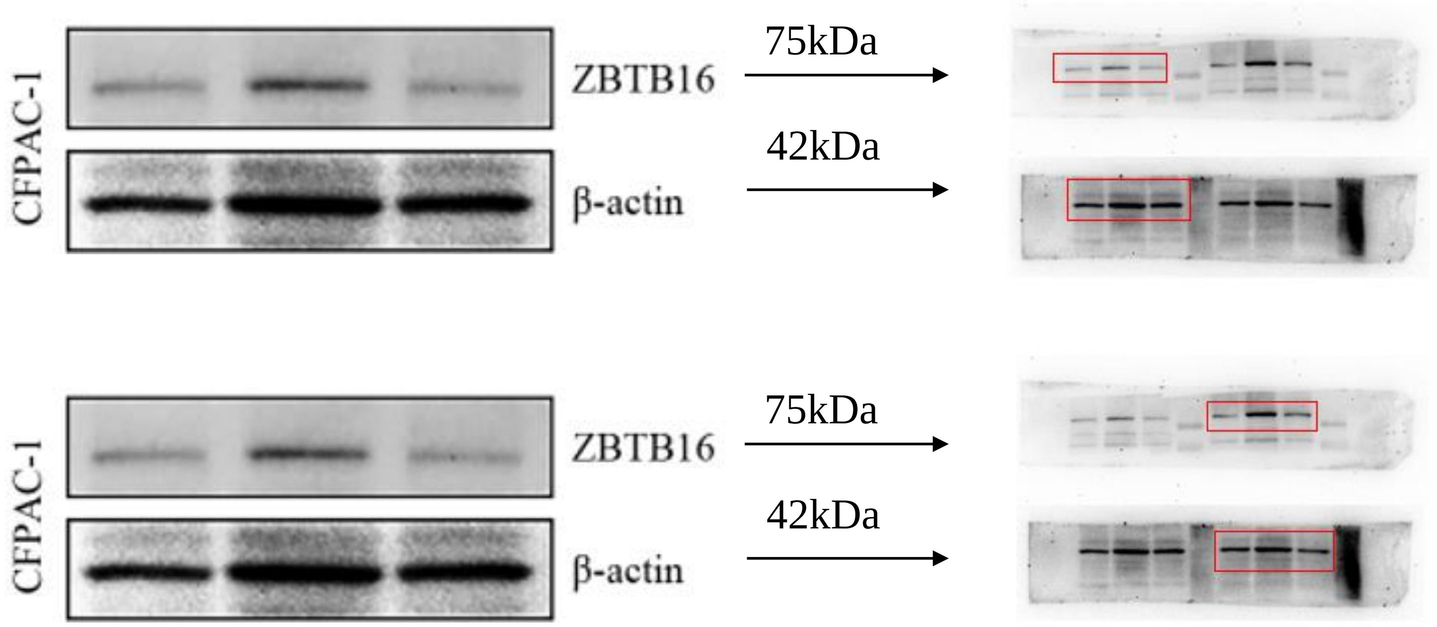

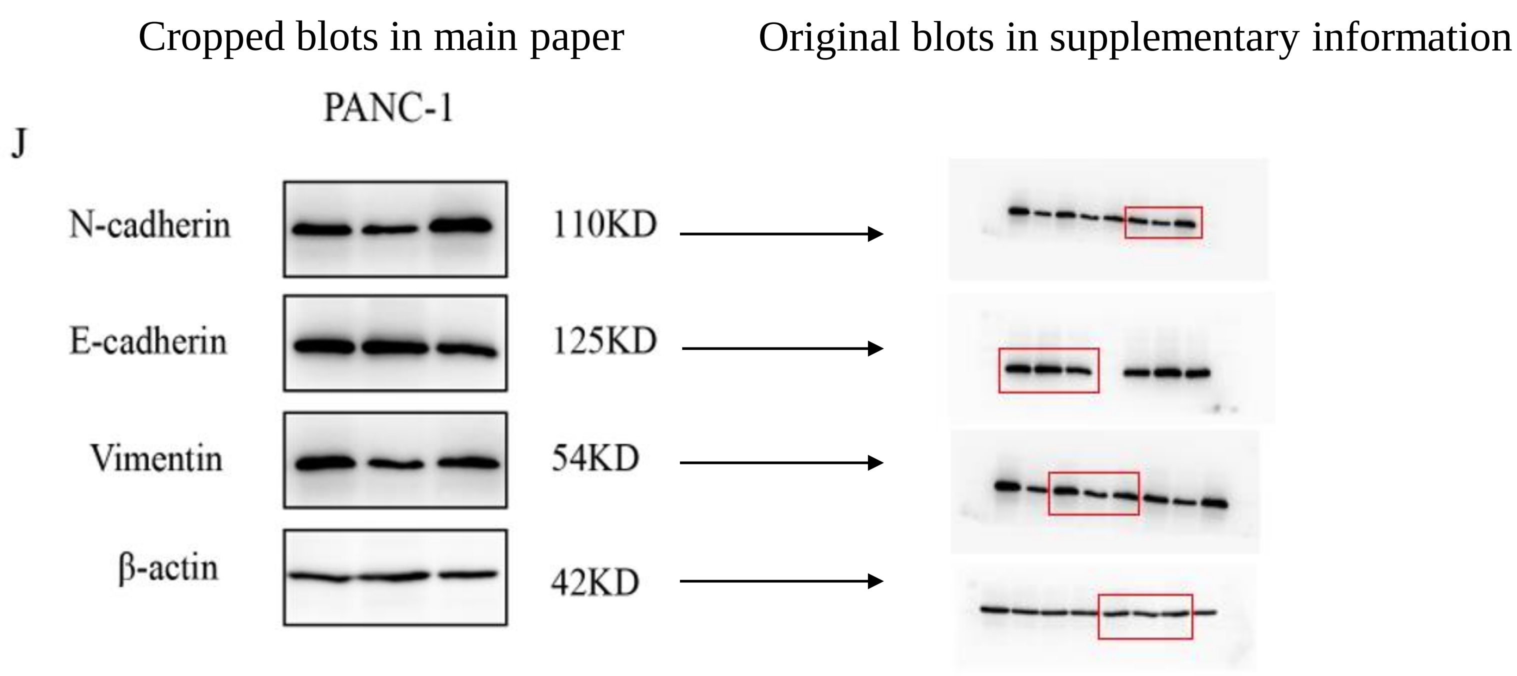
Cropped blots in main paper
Original blots in supplementary information
PANC-1
J
N-cadherin
E-cadherin
Vimentin
β-actin
110KD
125KD
54KD
42KD

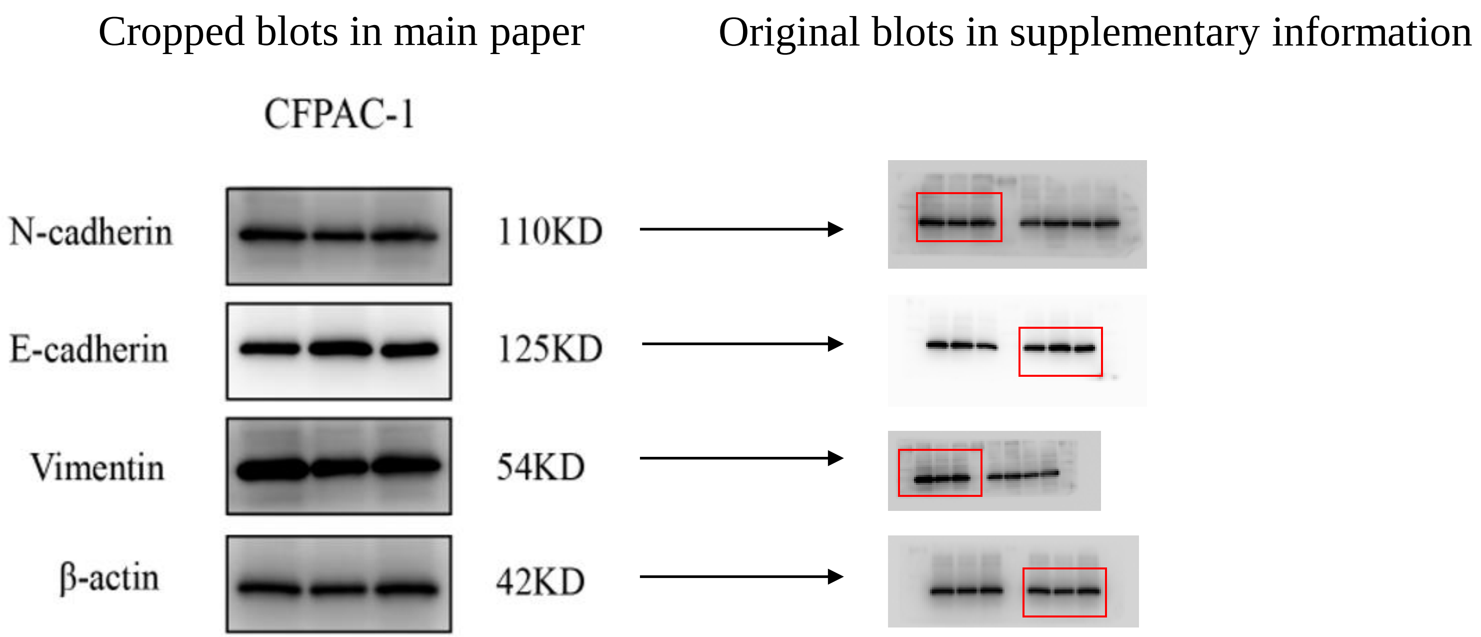
Cropped blots in main paper
Original blots in supplementary information
CFPAC-1
N-cadherin
110KD
E-cadherin
125KD
Vimentin
54KD
β-actin
42KD
